# Understanding the Impact of Vitamin B Supplements on a Saudi Population

**DOI:** 10.7759/cureus.50626

**Published:** 2023-12-16

**Authors:** Mohammad Othman, Mahdi Kanjo, Taha Tasji, Mohammed Rushan, Abdulrahman K Tasji, Abdulellah K Tasji, Wed K Tasji, Montaha K Tasji, Basil M Othman, Talah Tasji

**Affiliations:** 1 Clinical Sciences, Fakeeh College for Medical Sciences, Jeddah, SAU; 2 Neurology, Dr Soliman Fakeeh Hospital, Jeddah, SAU; 3 Medicine, Fakeeh College for Medical Sciences, Jeddah, SAU; 4 Medicine, Taibah University, Madinah, SAU; 5 Medicine, College of Medicine, King Saud Bin Abdulaziz University for Health Sciences, Jeddah, SAU

**Keywords:** gastric upset, erectile dysfunction, sleep pattern, bmi, energy, supplements, vitamin b

## Abstract

Introduction

B vitamins help generate energy within cells. A significant portion of populations in developed countries suffer a deficiency in one or more B vitamins. This study assesses the use of vitamin B supplements and their effects.

Methodology

This cross-sectional study was conducted using public participants in Saudi Arabia. Participants from all over Saudi Arabia were recruited through self-conducted surveys to study the effects of using vitamin B supplements on appetite, BMI, energy, and sleep, and to identify any side effects in participants. Inclusion criteria included age (18 years or older) and use of vitamin B supplements. Children, pregnant women, adults who had never used vitamin B, and those not willing to participate in the study were excluded.

Results

In total, 1,521 adults were recruited. Most of the participants were young Saudi Females. While taking vitamin B supplements, a minority of participants complained of mild gastrointestinal upset, but a significant proportion experienced no side effects. In this study, a significant proportion of participants experienced an increase in appetite, which was associated with a significant increase in BMI after taking vitamin B supplements. This study also explored increases in energy, which were significant and associated with significant increases in sleeping time. Male participants in the present study noticed a significant increase in erectile dysfunction (ED).

Conclusions

This study found significant effects of vitamin B supplements on BMI, appetite, energy, and sleep, as well as an increase in ED in male participants. More studies are needed to further explore these findings.

## Introduction

Vitamins are a group of substances that are essential for normal physiological function [1]. They are not synthesized by the body but can be obtained by diet [2]. There are 13 vitamins, some are fat-soluble (vitamins A, D, E, and K) while others are water-soluble (vitamin C and the eight B vitamins: thiamine (B1), riboflavin (B2), niacin (B3), pantothenic acid (B5), pyridoxine (B6), biotin (B7), folate (B9), and cobalamin (B12)). The B vitamins are grouped by water solubility and their inter-cellular functions [1-3]. B vitamins are generally synthesized by plants; however, vitamin B12 is produced by bacteria [4]. A vitamin supplement that contains nearly all eight B vitamins is referred to as vitamin B complex [3,4].

Although B vitamins are generally absorbed by the small intestine, bacterial B vitamins are produced and absorbed in the colon [5]. The body absorbs vitamin B12 from food in a two-step process. Hydrochloric acid in the stomach frees vitamin B12, and the vitamin then combines with a natural factor protein from the stomach. Both are absorbed together. Vitamin B12 in supplements is not attached to the natural factor protein, so people who cannot produce this natural factor, such as those with pernicious anemia, have trouble absorbing vitamin B12 from foods and supplements. They therefore should receive vitamin B12 intravenously to prevent deficiency [1,5,6].

B vitamins are involved in every aspect of generating energy within cells. Deficiency in any of the B vitamins will thus have negative consequences [1,3,5-8]. For example, vitamins B1, B2, B3, and B5 are essential co-enzymes in mitochondrial aerobic respiration and cellular energy production via their direct role in the citric acid cycle to produce adenosine triphosphate (ATP). Furthermore, B1, B7, and B12 play an essential part in the mitochondrial metabolism of glucose, fatty acids, and amino acids, thus contributing substrates to the citric acid cycle [1,5].

Substantial evidence suggests that a large portion of the populations in developed countries are suffering deficiency or borderline deficiency in one or more B vitamins. Such deficiency may have several negative health consequences, including lower-than-optimal brain function [1,9,10]. In addition, vitamin B deficiency has been associated with peripheral neuropathy (PN) [11,12]. Other studies have claimed that vitamin B may treat depression, but further analysis of these studies has indicated that this effect is merely protective and only present in older adults [1,4,13-16]. Other studies have demonstrated that taking vitamin B alongside either vitamin D or antidepressants may help prevent or lower the effects of depression in elderly patients; otherwise, vitamin B alone does not affect depression [1,4,13-17].

Vitamin B levels may be inversely related to sleep duration. Increasing levels of vitamin B improves sleep quality in insomnia patients but causes a decrease in sleep duration. Although naturally occurring levels of B12 are not associated with sleep disturbance, vitamin B supplements may be associated [13,18-25]. Furthermore, research dating to the 1960s has found that vitamin B causes an increase in body fat and, consequently, an increase in body mass index (BMI) and insulin resistance. These results were associated with both supplemental vitamin B and foods fortified with vitamin B [2,6,8,26-35].

Most of the studies on vitamin B concentrated mainly on one or two effects of using the supplements but none looked at these effects from all sides. This study assesses the association between vitamin B supplementation and BMI changes and explores the effects of vitamin B supplementation on sleep and mood changes. Side effects or complications related to the use of vitamin B supplementation are also examined.

## Materials and methods

This cross-sectional study was conducted using Twitter and WhatsApp in Saudi Arabia. Participants were recruited through a self-conducted survey. Inclusion criteria included a minimum age of 18 years, use of vitamin B, and willingness to participate. Children, pregnant women, people who had never used vitamin B, and those unwilling to participate were excluded. All participants received a simple questionnaire about their demographics, intake of vitamin B, and effects of the supplement on their health. Height data were collected and rounded to the nearest 0.1 cm. Weight data were also collected and rounded to the nearest 0.1 kg.

Following ethics requirements, participants’ privacy and confidentiality were protected by anonymizing data. The data were stored on a password-protected computer that only the principal researcher could access. Questionnaire data included the following: demographic information (age, nationality, education, occupation, gender, and presence of any chronic diseases); duration of vitamin B use; type of vitamin B used; method of intake; and changes in weight, appetite, mood, and sleep (Appendices). Ethical approval was obtained from the ethics committee of Fakeeh College for Medical Sciences (FCMS; ethical approval number 499-IRB-2023).

The sample size required for this research was calculated to be 148 if calculated for vitamin B9 [30], 584 if calculated for vitamin B3 [33], and 796 if calculated for vitamin B12 [33]. The sample size was calculated using the OpenEpi program (https://www.openepi.com/SampleSize). Because of the greater sample sizing, vitamin B12 was used as the main focus of this research for the sample size calculation. Extra participants were taken to represent non-responders (dropouts). Therefore, this study planned to recruit a minimum of 1000 adults. Data were managed and analyzed using Statistical Package for Social Sciences version 28 (IBM Corp., Armonk, NY, USA) [36], and the threshold for statistical significance was set to p ≤ 0.05. A paired sample student’s t-test was used to evaluate differences in means between groups for continuous variables. For categorical data, a chi-square test was used to assess differences in proportions across categories. Descriptive statistics were used whenever possible. These included qualitative variables calculated using frequency and percentage.

## Results

The questionnaire used in this study was promoted on Twitter and WhatsApp in Saudi Arabia for one month. In total, 1,521 adults responded to all questions and were included in the data analysis. Most of the participants were Saudi (1339, 88%) and female (1131, 74.4%). Participants were mainly young adults 18-25 years old (1040, 68.3%) and 26-35 years old (260, 17.1%); only 221, 14.6% were older than 35 years old. Most of the participants had a bachelor’s degree (1066, 70.1%) and were employed (871, 57.3%). Since most of the participants were young, the presence of chronic diseases was minimal (266, 17.1%) and the rest of the participants had no chronic diseases (1261, 82.9%). Characteristics of the participants are displayed in Table 1.

**Table 1:** Participant characteristics The data have been represented as N and (%).

	Total number of participants: 1,521	N (%)
Sex	Male	390 (25.6%)
	Female	1,131 (74.4%)
Nationality	Saudi	1,339 (88%)
	Non-Saudi	182 (12%)
Age (years)	18–25	1,040 (68.3%)
	26–35	260 (17.1%)
	36–45	117 (7.7%)
	46–55	65 (4.3%)
	Above 55	39 (2.6%)
Education	High school	325 (21.4%)
	Bachelor’s degree	1,066 (70.1%)
	Postgraduate degree	130 (8.5%)
Occupation	Student	429 (28.2%)
	Employed	871 (57.3%)
	Unemployed	195 (12.8%)
	Retired	26 (1.7%)
Presence of chronic diseases	None	1261 (82.9%)
	Diabetes mellites	117 (7.6%)
	Hypertension	78 (5.1%)
	Asthma	58 (3.6%)
	Other	13 (0.8%)

Characteristics of vitamin B use are included in Table 2. Use of vitamin B complex was the highest, followed by vitamin B12 (897, 59% and 533, 35%, respectively). Additionally, oral tablets were the most common method of consuming vitamin B (1378, 90.6%). Furthermore, using the supplement for 6-12 months was most common (793, 52.1%), followed by use for over 12 months (351, 23.1%).

**Table 2:** Vitamin B use by participants The data have been represented as N and (%).

	Total number of participants: 1,521	N (%)
Types of vitamin B	B complex	897 (59%)
	B12	533 (35%)
	B3	39 (2.6%)
	B1	52 (3.4%)
Mode	Oral tablets	1,378 (90.6%)
	Injections	143 (9.4%)
Duration of intake	1–3 months	143 (9.4%)
	3–6 months	234 (15.4%)
	6–12 months	793 (52.1%)
	More than 12 months	351 (23.1%)

Turning to the side effects of vitamin B supplements, there were minor complaints of mild gastric upset (312, 20.5%), but this difference was significant (p = 0.03). Similarly, many participants exhibited an increase in appetite (1326, 87.2%) and a change in BMI before and after the use of supplements (1378, 90.6%). Both were significant (p = 0.03). Additionally, there was an increase in energy in participants (975, 64.1%), as well as changes in sleep duration and sleep patterns (1014, 66.7%) and mood (1040, 68.4%). All these changes were significant (p =0.03; Table 3).

**Table 3:** Effects of using vitamin B The data have been represented as N and (%); the p-value is considered significant if p<0.05.

	Total number of participants: 1,521	N (%)	p
Minor side effects	None	1,209 (79.5%)	0.03
	Gastric upset	312 (20.5%)	
Increased appetite	Yes	1,326 (87.2%)	0.03
	No	195 (12.8%)	
Changes in BMI	Yes	1,378 (90.6%)	0.03
	No	143 (9.4%)	
Increased energy	Yes	975 (64.1%)	0.03
	No	546 (35.9%)	
Changes in sleep	Yes	1,014 (66.7%)	0.03
	No	507 (33.3%)	
Changes in mood	Yes	1,040 (68.4%)	0.03
	No	481 (31.6%)	

To evaluate changes in BMI, World Health Organization (WHO) BMI categories were used. BMI was compared before and after the use of vitamin B supplements. The difference between categories was not significant (p = 0.3; Table 4).

**Table 4:** Comparison of BMI categories before and after vitamin B use The data have been represented as N and (%); the p-value is considered significant if p<0.05.

BMI categories	BMI before vitamin B {N (%)}	BMI after vitamin B {N (%)}	p	
Below 18.5	791 (52%)	79 (5.2%)	0.025	
18.5–24.9	202 (13.3%)	335 (22%)	0.025	
25–29.9	82 (5.4%)	415 (27.3%)	0.025	
30–34.9	316 (20.8%)	406 (26.7%)	0.025	
35–39.9	92 (6%)	191 (12.6%)	0.025	
40 and above	38 (2.5%)	95 (6.2%)	0.025	

This study also explored sleeping duration before and after the use of vitamin B supplements; changes were not significant (p = 0.5; Table 5).

**Table 5:** Comparison of sleeping time before and after vitamin B use The data have been represented as N and (%); the p-value is considered significant if p<0.05.

Duration of sleep (hours)	Before vitamin B {N (%)}	After vitamin B {N (%)}	p	
Less than 6 hours	78 (5.1%)	195 (12.8%)	0.025	
6–8 hours	967 (63.6%)	390 (25.6%)	0.025	
8–12 hours	453 (29.8%)	767 (50.4%)	0.025	
More than 12 hours	23 (1.5%)	169 (11.2%)	0.025	

Notably, men in the study complained of erectile dysfunction (ED). This symptom was present in 298 (76.4%) men, and this result was significant (p = 0.03; Table 6).

**Table 6:** Erectile dysfunction in men The data have been represented as N and (%); the p-value is considered significant if p<0.05.

Participant reply	Total number of male participants: 390 N (%)	p
Yes	298 (76.4%)	0.03
No	92 (23.6%)	

## Discussion

This study recruited 1,521 participants. Most of the participants were young Saudi females with a bachelor’s degree who were employed and had no chronic disease. Most participants had been using oral tablet vitamin B complex for at least 6-12 months (Table 2). A minority of the participants complained of mild gastrointestinal upset, but a significant proportion experienced no side effects. This finding is consistent with existing research: side effects of vitamin B intake are mainly mild nausea or vomiting but rarely reach hepatic toxicity [37].

In this study, a significant proportion of participants experienced a change in appetite, and this was accompanied by a significant change in participant BMI after taking vitamin B supplements. These findings are similar to two previous large randomized controlled trials, where a significant proportion of normal and underweight participants shifted to the overweight and obese BMI categories [8,31]. These increases in weight and the shift of participants toward obesity are mainly due to fast fat deposition all over the body and on the muscles [29,30,33-35]. In the present study, changes in BMI before and after the use of vitamin B were present but did not reach significance (Table 4). This non-significant result may be due to an underpowered study; moreover, changes in each BMI category were significant (p = .025), but overall changes in BMI were not. This finding is important and motivates future studies to explore changes in BMI related to vitamin B supplement use.

This study also explored increases in energy, which were significant and associated with a significant increase in sleeping time. This finding has been present in many previous studies [7,21,23]. However, upon a detailed exploration of existing sleep data, it becomes apparent that the real rest time decreased [17-20,22,35]. These findings were explored in the present study, and though participants' sleep time increased such changes were not statistically significant. However, when comparing pre- and post-vitamin B supplement use for specific sleep duration categories, changes were significant. As with the BMI data, these changes did not reach significance at the group level (Table 5). In previous studies, the increase in sleeping time is obvious, but sleeping patterns were affected and resulted in decreased resting time [17-19,35]. This finding requires further exploration - future studies should assess changes in sleeping time and patterns related to the intake of vitamin B supplements.

On reviewing the literature, patterns start to appear. Participants who take vitamin B notice an increase in appetite and weight gain, as evidenced by increased BMI. Increases in sleeping time but less rest time affect participants' moods and are accompanied by an unusual increase in energy. These outcomes dispose participants of depression and promote food intake, leading to an increase in BMI with faster fat deposition [1,2,9,14,20,28,30,33].

Male participants in the present study noticed significant increases in ED. This finding has been studied extensively in previous research, and similar results were found in some studies [38,39]. On the contrary, some researchers found no relation between vitamin B and ED [40,41]. Noticeably, in studies that found no effect of vitamin B on ED, the use of vitamin B was only for short periods (less than 6 months [41]).

The main limitation of this study is its cross-sectional design, but the collection of so many participants over a short time is best done through social media in cross-sectional studies. Another limitation is that data on the nutritional habits of the participants were unavailable. These data were not collected to make the survey easier for social media users to complete but could have partially explained the mechanisms underlying some of the significant findings. In addition, as data on supplement consumption and outcomes were self-reported, a reporting bias might exist: individuals may not have accurately reported their real vitamin intake. However, such a bias is due to underreporting by participants. Lastly, the reason for vitamin B supplement use was not explored.

## Conclusions

This study presented important findings including that vitamin B supplements may increase weight by increasing fat deposition. Vitamin B may also increase sleeping time and energy, but the quality of sleep and rest time should be explored more. Vitamin B supplements are associated with mild gastric upset, but, more importantly, may cause ED in men. More research (randomized controlled trials and systematic reviews with meta-analysis) should be done on vitamin B and its effects on the body, and future studies should be large enough to explore each difference's potential mechanisms.

## Appendices

**Table 7:** Questionnaire used in the study

Age in years	18-24		25-35							36-45									46+			
Nationality	Saudi										Non-Saudi											
Occupation																						
Education	Primary				Intermediate											Secondary						
	Bachelor				Postgraduate																	
Have you ever used vitamin B complex?						For how long																
Which form?	Oral												Injections									
Have you ever had your Vit B level measured?			Do not know												Yes						No	
What was your weight before taking Vit B?							Your height											BMI				
What is your weight now?								BMI now														
Have you noticed any change in appetite after using Vit B?																						
Have you noticed any change in sleep after using Vit B?																						
How many hours do you sleep every night?																						
Have you noticed any change in energy after using Vit B?																						
Have you noticed any change in mood after using Vit B?																						
Have you noticed any change in personality after using Vit B?																						
Have you noticed any other changes or complications after using Vit B?																						
Do you use any medication for long period?		Yes		No					Do not know					Name of drug								
Do you have any chronic diseases?		Yes		No					Do not know					Which disease								
